# Proteomic Analysis of Plasma from California Sea Lions (*Zalophus californianus*) Reveals Apolipoprotein E as a Candidate Biomarker of Chronic Domoic Acid Toxicosis

**DOI:** 10.1371/journal.pone.0123295

**Published:** 2015-04-28

**Authors:** Benjamin A. Neely, Jason A. Ferrante, J. Mauro Chaves, Jennifer L. Soper, Jonas S. Almeida, John M. Arthur, Frances M. D. Gulland, Michael G. Janech

**Affiliations:** 1 Department of Medicine, Division of Nephrology, Medical University of South Carolina, Charleston, SC, United States of America; 2 Grice Marine Laboratory, College of Charleston, Charleston, SC, United States of America; 3 The Marine Mammal Center, Sausalito, CA, United States of America; 4 Department of Biomedical Informatics, Stony Brook University, Long Island, NY, United States of America; 5 Research Service, Ralph H. Johnson VA Medical Center, Charleston, SC, United States of America; Sonoma State University, UNITED STATES

## Abstract

Domoic acid toxicosis (DAT) in California sea lions (*Zalophus californianus*) is caused by exposure to the marine biotoxin domoic acid and has been linked to massive stranding events and mortality. Diagnosis is based on clinical signs in addition to the presence of domoic acid in body fluids. Chronic DAT further is characterized by reoccurring seizures progressing to *status epilepticus*. Diagnosis of chronic DAT is often slow and problematic, and minimally invasive tests for DAT have been the focus of numerous recent biomarker studies. The goal of this study was to retrospectively profile plasma proteins in a population of sea lions with chronic DAT and those without DAT using two dimensional gel electrophoresis to discover whether individual, multiple, or combinations of protein and clinical data could be utilized to identify sea lions with DAT. Using a training set of 32 sea lion sera, 20 proteins and their isoforms were identified that were significantly different between the two groups (*p*<0.05). Interestingly, 11 apolipoprotein E (ApoE) charge forms were decreased in DAT samples, indicating that ApoE charge form distributions may be important in the progression of DAT. In order to develop a classifier of chronic DAT, an independent blinded test set of 20 sea lions, seven with chronic DAT, was used to validate models utilizing ApoE charge forms and eosinophil counts. The resulting support vector machine had high sensitivity (85.7% with 92.3% negative predictive value) and high specificity (92.3% with 85.7% positive predictive value). These results suggest that ApoE and eosinophil counts along with machine learning can perform as a robust and accurate tool to diagnose chronic DAT. Although this analysis is specifically focused on blood biomarkers and routine clinical data, the results demonstrate promise for future studies combining additional variables in multidimensional space to create robust classifiers.

## Introduction

Domoic acid toxicosis (DAT), first diagnosed in California sea lions (*Zalophus californianus*) in 1998, is caused by the ingestion of toxin originating from diatoms of the genera *Pseudo-nitzschiza* [1–3]. Mass stranding events of California sea lions along the west coast of the United States ascribed to domoic acid ingestion have been reported repeatedly since 1998 [4]. Sea lions with DAT present with ataxia and seizures, often times resulting in death [5, 6]. In addition to these non-specific clinical signs, the diagnosis of DAT is based on detection of domoic acid in body fluids, although the rapid clearance of domoic acid from the body can be confounding [7]. Diagnosis of DAT may be confirmed post-mortem (*e*.*g*., hippocampal atrophy) [6, 8]. The pathology of DAT results from hippocampal insults due to excessive neuroexcitation which leads to epileptiform seizures [9–13] primarily through activation of the AMPA and kainate receptors [14–16], followed by subsequent activation of NMDA receptors possibly through arginine release [17–19]. Although neurotoxic, domoic acid has also been implicated in the development of cardiomyopathy [20] and acute kidney injury [21] suggesting the disruption of multiple organ systems. Affected individuals can be characterized along a continuum of toxicosis ranging from acute to chronic. Acute DAT comprises the initial time phase of hippocampal damage, from which sea lions can recover, while chronic DAT is generally defined as recurrent seizures progressing to *status epilepticus* [5, 22].

Minimally-invasive tests for DAT have been the focus of numerous recent biomarker studies with the goal to achieve a robust and accurate method to discriminate sea lions with DAT from animals stranded due to other etiologies. To overcome the issue of rapid domoic acid clearance, an indirect enzyme-linked immunosorbant assay (ELISA) has been reported that utilizes circulating antibodies to domoic acid to discriminate between wild sea lions exposed to domoic acid and unexposed captive sea lions, suggesting that domoic acid exposure can be detected well after an exposure event [23]. Eosinophilia also appears to be a clinical sign of domoic acid exposure and possibly a general response to domoic acid in marine mammals [5, 24–26]. A prospective cross-sectional study suggested that eosinophil count alone, or possibly combined with low cortisol levels, may provide a reasonable classifier of acute or chronic DAT in California sea lions [27]. Serum peptide profiles measured by matrix-assisted laser desorption ionization time-of-flight (MALDI-TOF) mass spectrometry have been investigated as classifiers of acute DAT [26]. Although classifying peptides were not identified, combinations of features incorporated in feed-forward artificial neural networks provided predictive models that outperformed individual peptide features in an external validation test. However, combinations of clinical and peptide data together were not reported to evaluate whether model performance could be enhanced.

The intent of this study was to profile plasma proteins in sea lions with chronic DAT and those without DAT using two dimensional gel electrophoresis (2D-GE) in effort to discover whether individual, multiple, or combinations of protein and clinical data could be utilized to classify sea lions with DAT. To this end, plasma was collected from sea lions with chronic DAT, stranded sea lions without DAT, or sea lions without DAT that had recovered from an ailment and were soon to be released back into the wild. Herein, we present data suggesting that low levels of a charge-form of apolipoprotein E (ApoE) together with eosinophil counts can be a good discriminative test for the diagnosis of chronic DAT in sea lions.

## Materials and Methods

### Plasma collection and storage

Samples were collected by The Marine Mammal Center (TMMC; Sausalito, CA) using TMMC’s Internal Animal Care and Use Committee approved protocols (number 2007–2) under the National Fisheries Service permit number 932-1489-00 and Marine Mammal Protection Act permit number 932-1905-00 as part of a standard clinical care regime. Blood samples were collected in Na-citrate tubes, centrifuged at 3,000 rpm for 10 min, and plasma was immediately transferred to a cryovial and stored at -80°C. Less than 2 h passed between collection and storage at -80°C. All samples were thawed at the Medical University of South Carolina at 37°C for one min, then placed on ice and 60 to 110 μL aliquots were stored at -80°C. Hematology data were included for comparison if collected the same day as the plasma. Complete blood count (CBC) analyses included total white blood cell count (WBC) with differential (segmented neutrophils, Seg; band cells, Band; lymphocytes, Lymph; monocytes, Mono; eosinophils, Eos), red blood cell count (RBC), hemoglobin (HGB), hematocrit (HCT), platelet (PLT), mean corpuscular volume (MCV), mean corpuscular hemoglobin (MCH), mean corpuscular hemoglobin concentration (MCHC), red cell distribution width (RDW), and mean platelet volume (MPV).

### Inclusion criteria

Individuals used in the training set were selected retrospectively from individuals admitted to TMMC between 2007 and 2011 suffering from chronic DAT or asymptomatic for DAT. Sea lions with DAT are referred to as the DAT group. Sea lions that did not have DAT are referred to as the non-DAT group. Within the non-DAT group some sea lions had “recovered” from an ailment or others had died in treatment; therefore, were labeled as non-“recovered”. Recovered individuals had blood drawn within a week of release as part of routine clinical care. Of the DAT group, 8 of 12 died during treatment or were euthanized. Of the non-DAT non-“recovered” animals, all died during treatment or were euthanized. Of the non-DAT “recovered” individuals, none died during treatment or were euthanized. Plasma samples were used if the draw date was within seven days of admission or within seven days of release, with the exception of one “recovered” individual (CSL-7671) which was 68 days from admission but 19 days from release. Chronic DAT was defined based on criteria described by Goldstein *et al*. [22] and Gulland *et al*. [27] as well as with available brain histology (*i*.*e*., hippocampal atrophy indicated chronic DAT [6]), previous stranding data, and/or if seizing occurred within a week after stopping medication to treat acute toxicosis. Individuals in the independent test set were selected from plasma taken at admission only (*e*.*g*., there were no “recovered” individuals in the qualification set) and outcomes were blinded to investigators until analysis and predictions were made.

### Exclusion criteria

Plasma from third trimester pregnant individuals or those from individuals with massive traumas were excluded from the training and qualification sets. Non-DAT individuals were excluded if they later presented with DAT. Chronic DAT individuals were not allowed to have leptospirosis or other seriously confounding etiologies.

### Experimental design

A training set of plasma samples were processed and analyzed for biomarkers, followed by an independent blinded test set that was used to qualify biomarker performance. The training set (n = 32) was comprised of plasma from chronic DAT (n = 12) and non-DAT (n = 20). The non-DAT group contained 10 non-recovered and 10 recovered individuals. There was an effort made to frequency match based on age class as defined in [28] and sex. The independent test set (n = 20) was blinded to Medical University of South Carolina personnel processing the samples and analyzing the data. Test set samples were shipped, processed and analyzed independently of the training set. This test set was chosen using the same criteria as the training set and an attempt was made to match the age and gender distribution of the test set to the training set. The non-DAT etiology distribution was allowed to vary, but reflects the stranding population at The Marine Mammal Center during the time of the study.

### Plasma enrichment

Immediately prior to enrichment, frozen plasma samples were rapidly thawed at 37°C, placed on ice, and protein concentrations were determined by the Bio-Rad Protein assay (Hercules, CA) which is based on the Bradford method. Protein concentrations were normalized to ≤50 mg/mL by diluting with PBS if necessary. Lower abundance plasma proteins were enriched by depleting higher abundance proteins, thus decreasing the large dynamic range of protein concentrations therein, and ProteoMinter protein enrichment was utilized based on previous research comparing enrichment strategies [29]. Enrichment of low abundance proteins was accomplished using the ProteoMiner Protein Enrichment Small-Capacity Kit (Bio-Rad) following a variation of the methods provided by the manufacturer. Briefly, the storage solution was removed from the ProteoMiner spin columns by centrifugation (1,000 *g* for 1 min). Next, the spin columns were washed once with deionized water and twice with wash buffer (10 mM NaH_2_PO_4_, pH 7.4) by gently rotating the capped column and solution for 5 min followed by centrifugation (1,000 *g* for 1 min). For each sample, 10% (v/v) plasma preparation buffer (1 M sodium citrate, 20 mM HEPES, pH 7.4) was added to 10 mg of sample and loaded on a capped spin column. The spin column containing the sample and the ProteoMiner beads was gently rotated for 3 h. The flow through was discarded by centrifugation (1,000 *g* for 1 min) and the column was washed three times with wash buffer and twice with deionized water. Finally, 100 μL of boiling elution buffer [4% sodium dodecyl sulfate (SDS), 25 mM dithiothreitol (DTT)] was added to the ProteoMiner spin column and lightly vortexed for 15 min followed by centrifugation (1,000 *g* 1 min). The process was repeated twice for each plasma and the eluents were combined.

### Two-dimensional gel electrophoresis

The enriched samples were acetone precipitated. Briefly, 5 volumes (v/v) cold acetone (stored at -20°C) was added to the enriched samples and incubated at -20°C for 1 h followed by centrifugation (15,000 *g* for 10 min). The supernatant was discarded and the pellets were dried. Each precipitated sample was re-suspended in rehydration buffer (50 mM DTT, 7 M urea, 2 M thiourea, 4% CHAPS, 1% ampholytes, 1.2% DeStreak) and incubated at 4°C overnight. A Bradford assay determined the protein concentration of the re-suspended samples. Next, rehydration buffer was added to 350 μg of the re-suspended sample to reach a total volume of 300 μL. Ultra-centrifugation (100,000 *g* for 30 min) sedimented particulates from the samples and the supernatant was placed on 17cm pH 4 to 7 IPG strips (Bio-Rad). The strips were actively rehydrated at 50V overnight on a Protean IEF Cell (Bio-Rad) and desalted at 300V for 4 h. Finally, the strips were focused at 10,000 V/hr for a total of 60,000 V followed by a hold step at 500 V.

The samples were reduced by washing the strips three times with equilibration buffer (375 mM Tris pH 8.8, 6M urea, 2% SDS, 20% glycerol) containing 2% (w/v) DTT for 10 min each time and then alkylated by washing three times with equilibration buffer containing 2.5% (w/v) iodoacetamide (IAA) for 10 min each time. Next, each strip was washed with TGS running buffer (25 mM Tris, pH 8.3, 192 mM glycine, 0.1% SDS) and loaded onto an SDS gel (10% acrylamide, 375 mM Tris, pH 8.8, 0.1% SDS, 0.1% ammonium persulfate, 0.1% tetramethylethylenediamine). A sealing solution (TGS buffer containing 0.5% agarose and a trace amount of bromophenol blue) was carefully poured on top of the strips before running them. The gel electrophoresis was run at 16 mA per gel for 30 min, followed by 24 mA per gel for 30 min and finally 45 mA per gel until the dye front had reached the bottom.

The gels were fixed with a solution containing 10% methanol and 7% acetic acid for 30 min, rinsed with deionized water, and stained overnight with SYPRO Ruby (Bio-Rad). After rinsing with deionized water, the gels were de-stained with a solution containing 10% methanol and 7% acetic acid for 30 min, followed by three rinses with deionized water.

### Spot identification by tandem mass spectrometry

Gels were scanned on a Molecular Imager FX Pro (Bio-Rad) using PDQuest 2-D (v. 7.4; Bio-Rad) and analyzed using Progenesis SameSpots (v. 3.3; Nonlinear). Protein spots of interest were aligned and excised with a BioRad proteome works spot cutter. Gel pieces are washed with 50:50 acetonitrile (ACN) ammonium bicarbonate (AmBic) three times for 30 min each time, then allowed to dry at 37°C for 30 min. Next, 250 ng of trypsin gold (Promega) was added and incubated at 37°C overnight. Peptide solutions were removed and the gel pieces were washed twice with 50% ACN, 2% formic acid for 30 min each time. The collected supernatents were dried in a speedvac. The resulting pellet was reconstituted in 20 μL of mobile phase A (MPA; 2% ACN, 0.1% formic acid).

Peptides (10 μl) were injected onto a 100 μm × 1 cm C18 (100 Å with 5-mm particles) trap column (Acclaim PepMap 100; Thermo Fisher Scientific) at 5 μL/min for 10 min with 100% MPA, and separated on a 75 μm × 15 cm C18 (300 Å with 3-μm particles) analytical column (Acclaim PepMap 100; Thermo Fisher Scientific). Reverse phase separation at 300 nL/min was performed with a gradient of 3% to 30% mobile phase B (MPB; 95% ACN, 0.1% formic acid) over 14 min followed by 30% to 85% MPB over 9 min on a 2D+ NanoLC system (Eksigent, Dublin, CA). The LC was interfaced to a TripleTOF 5600 System (AB Sciex, Foster City, CA) with a nanospray source. Source temp was set at 120°C, and source voltage was set at 2500 V. The declustering potential was set at 110 V. The instrument was run in positive ion instrument dependent acquisition mode with precursor ion scans for 250 ms (300 to 1250 *m/z*) with up to 20 product ion scans of 150 ms for precursors between 300 to 1250 *m/z*, exceeding 125 cps, and had a 2+ to 5+ charge state. Rolling collision energy was used to fragment precursors. Product ion data was collected for a mass range between 100 to 1600 *m/z*.

Resulting raw files were converted to peak lists using the AB SCIEX MS Data Converter (v. 1.1 beta, July 2011). Protein identifications were made using Mascot (v. 2.3.02) by searching an investigator-selected combination of mammalian databases. This database was a combined UniProt SwissProt, SwissProt varsplic, and TrEMBL database (release 2012_3) using the following species (with common name and UniProt taxonomy identifier in parentheses): *Ailuropoda melanoleuca* (Giant Panda; 9646), *Canis lupus familiaris* (Dog; 9615), *Homo sapiens* (Human; 9606), *Sus scrofa* (Pig; 9823), *Bos taurus* (Bovine; 9913), *Mus musculus* (Mouse; 10090), and *Zalophus californianus* (California sea lion; 9704). The final parsed database was comprised of 44,953 entries from SwissProt, 282,269 entries from TrEMBL, and 23,966 entries from SwissProt varsplic. Additionally, this database contained the common Repository of Adventitious Proteins database (cRAP; 2012.01.01; 107 sequences; the Global Proteome Machine). The data was searched using the following parameters: trypsin was selected as the enzyme and two missed cleavages were allowed; carbamidomethylation (C) was specified as a fixed modification; deamidation (NQ) and oxidation (M) were specified as variable modifications; a precursor tolerance of 20 ppm and fragment ion tolerance of 0.5 Da; instrument type was ESI-QUAD-TOF. The mass spectrometry proteomics data have been deposited to the ProteomeXchange Consortium (http://proteomecentral.proteomexchange.org) via the PRIDE partner repository [30] with the dataset identifier PXD001606. Identifications which mapped to uncharacterized protein entries were assigned a protein name if possible by using Blastp on the UniProtKB.

### Data analysis tools and approach

Hematological parameters were compared between DAT and non-DAT groups using a Wilcoxon rank-sum test with normal approximation (Matlab; R2013a; Mathworks, Natick, MA). Spot intensity measurements were exported from Progenesis SameSpots for further analysis. Unsupervised hierarchal clustering analysis was performed using Matlab. Spot data was standardized within spots by calculating the mean value of the 32 individuals, and determining the standard deviation units around this mean. This was used to generate a hierarchical tree by calculating Euclidean pairwise distances, followed by clustering along patients (*e*.*g*., creating spot clustered data). Principle component analysis was performed using Matlab after first standardizing the data to have unit variances. Receiver operator characteristic curves were calculated using Matlab. If the median healthy value was greater than the median diseased, the curve was constructed as value less than threshold equaled disease outcome, otherwise value greater than equal threshold equaled disease outcome. Using the constructed ROC curve, two thresholds were calculated [26]: minimum mis-classified threshold (minMC) and optimum threshold (OpT). The minMC is the threshold that minimizes the proportion of false-negatives and false-positives. The OpT is the geometrically determined threshold which is the closest point to 100% sensitivity and 100% specificity which corresponds to the perfect test [31]. Additional machine learning algorithms (below) were employed using Matlab. Artificial neural networks (ANN) were trained by allowing each ANN to be pursued to an early stop criteria determined by internal testing using bootstrapped cross-validation with every seventh sample, in addition to screening for optimal topology. To generate an ensemble prediction from 101 trained ANNs, the output was aggregated by averaging the ANN output for each individual. A threshold was determined using the ensemble output from the training set to generate an ROC curve. This threshold was used to query the independent test set. This combined ANN (or ensemble ANN) approach is termed CANN_101_ herein. A categorical classification tree (CT) was grown using an exact search to determine best splits. A random forest (RF) was generated by first determining the optimum minimum leaf size and number of trees by finding the minimum out-of-bag-error (oobError) on the training set. The optimum model was comprised of 100 trees with minimum leaf size of 1 and had an oobError of 0.167. A *k*-nearest neighbor (*k-*NN) model was used to generate a classification model using euclidian distance metric without distance weighting and the nearest neighbor (*k* = 5) was chosen based on cross-validation loss in the training set. Lastly, a support vector machine (SVM) was trained, and a radial basis function (rbf) kernel was selected based on performance on the training set.

## Results

### Patient characteristics

An archived set of plasma samples drawn from sea lions admitted to the Marine Mammal Center was used to identify markers of chronic DAT. This training set was comprised of plasma from 32 sea lions with chronic DAT (n = 12) and non-DAT (n = 20; Table 1). There were no differences in gender or age class [28] between the DAT and non-DAT groups (*p* = 0.098, one-sided Fisher’s exact test; *p* = 0.836, chi-square test; respectively). Within the non-DAT group the major cause of stranding was leptospirosis, with equal distribution of cases between the non-DAT non-recovered and non-DAT recovered individuals (four in each). All of the non-DAT non-recovered samples were from sea lions that were later euthanized, whereas the non-DAT recovered samples were taken near time of release from the Marine Mammal Center. Of the hematological parameters measured between the groups, red blood cell count (RBC), hemogloblin (HGB) and eosinophil counts were significantly different between the groups (Table 2).

**Table 1:** Training set characteristics table.

	Total	DAT	Non-DAT
**Total**	32	12	20
male	14	3 (25%)	11 (55%)
female	18	9 (75%)	9 (45%)
**Age**			
*pup*	1	0 (0%)	1 (5%)
*yearling*	2	1 (8%)	1 (5%)
*juvenile*	7	2 (17%)	5 (25%)
*subadult*	9	3 (25%)	6 (30%)
*adult*	13	6 (50%)	7 (35%)
**Euthanasia/Death**	18	8 (67%)	10 (50%)
**Primary Etiology (non-DAT)**			
*leptospirosis*		-	8
*trauma*		-	2
*carcinoma*		-	3
*malnutrition*		-	6
*pneumonia*		-	1

Descriptive data of sea lions in the training set.

**Table 2:** Hematological parameters of the training set.

	DAT	non-DAT	
Parameter	Avg ± S.D. (n)	Avg ± S.D. (n)	*p*-value
WBC (10^3^/mm^3^)	13.4 ± 4.7 (n = 11)	17.0 ± 7.0 (n = 17)	0.095
RBC (10^6^/mm^3^)	5.0 ± 0.6 (n = 11)	4.2 ± 0.5 (n = 19)	0.002*
HGB (g/dL)	17.8 ± 2.0 (n = 11)	14.8 ± 1.8 (n = 19)	0.001*
HCT (%)	47.6 ± 12.5 (n = 11)	43.0 ± 5.9 (n = 19)	0.078
PLT (fL)	407.9 ± 163.1 (n = 11)	473.8 ± 188.7 (n = 18)	0.252
MCV (pg)	101.6 ± 6.1 (n = 11)	102.2 ± 4.7 (n = 19)	0.729
MCH (g/dL)	35.5 ± 1.7 (n = 11)	35.4 ± 1.7 (n = 19)	0.763
MCHC (10^3^/mm^3^)	35.3 ± 1.8 (n = 11)	34.6 ± 1.3 (n = 19)	0.438
RDW (%)	15.6 ± 0.8 (n = 11)	15.6 ± 1.1 (n = 19)	0.846
MPV (fL)	7.9 ± 1.0 (n = 11)	8.4 ± 1.0 (n = 19)	0.149
Seg (#/mm^3^)	8875 ± 4328 (n = 11)	12309 ± 7200 (n = 17)	0.221
Band (#/mm^3^)	57 ± 85 (n = 11)	1172 ± 2207 (n = 17)	0.063
Lymph (#/mm^3^)	3339 ± 1247 (n = 11)	2756 ± 1602 (n = 17)	0.371
Mono (#/mm^3^)	160 ± 175 (n = 11)	227 ± 458 (n = 17)	0.443
Eos (#/mm^3^)	936 ± 461 (n = 11)	545 ± 566 (n = 17)	0.024*

Blood data is based on analysis of blood drawn the same day as samples used for analysis. Complete blood count analyses of the 32 samples used in the training set are listed. Some parameters were not measured for all individuals and are indicated by ‘n’. Hematological parameters were compared between chronic domoic acid toxicosis (DAT) and non-DAT groups using a Wilcoxon rank-sum test with normal approximation, and significance (p<0.05) is highlighted by an ‘*’. *Abbreviations: WBC, white blood cell count; RBC, red blood cell count; HGB, hemoglobin; HCT, hematocrit; PLT, platelet; MCV, mean corpuscular volume; MCH, mean corpuscular hemoglobin; MCHC, mean corpuscular hemoglobin concentration; RDW, red cell distribution width; MPV, mean platelet volume; Seg, segmented neutrophils; Band, band cells; Lymph, lymphocytes; Mono, monocytes; Eos, eosinophils.*

### 2D gel and individual spot performance

**Fig 1:**
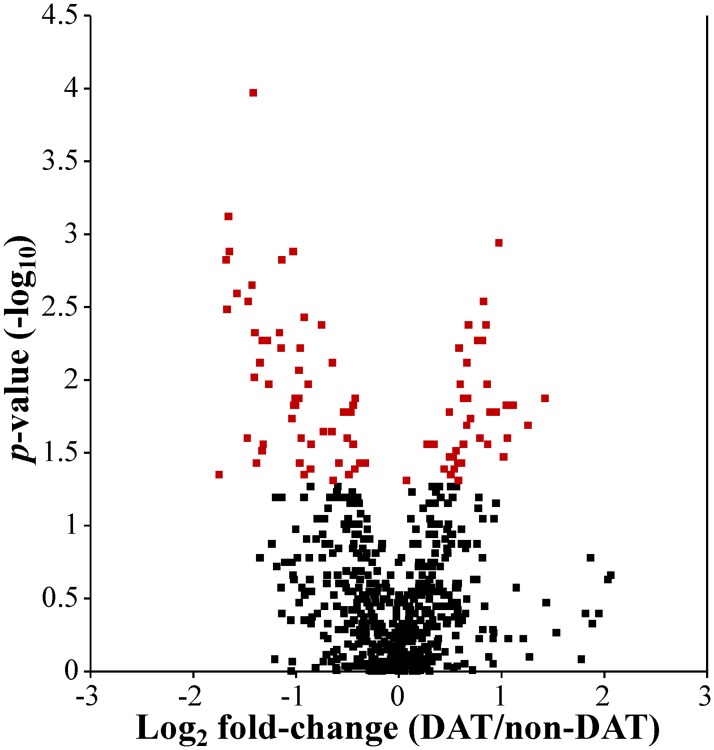
Volcano plot of all 618 spots in the training set. Spot intensities were compared between chronic DAT and non-DAT samples using a Wilcoxon rank sum test and the-log_10_ *p*-value and log_2_ fold-change (DAT/non-DAT) values were used to generate a volcano plot. Red squares indicate spots with a *p*<0.05 (n = 93).

**Fig 2:**
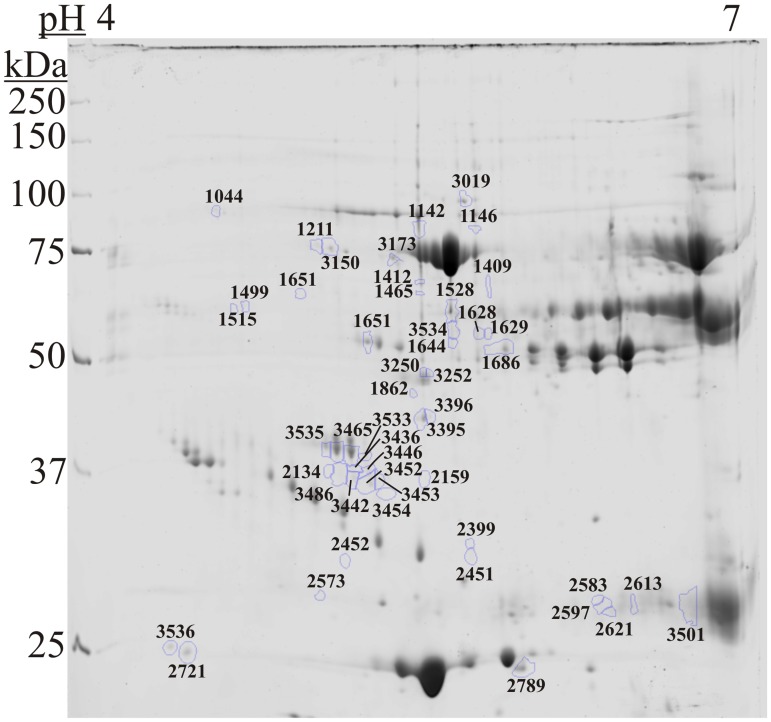
Two-dimensional gel electrophoresis. Depleted serum from 32 sea lions was separated using 2-dimensional gel electrophoresis. Shown is a representative gel with 49 spots of interest that were used for downstream analysis.

Using two-dimensional gel electrophoresis, 618 spots were selected and intensities were calculated for each of the 32 gels. All the spots were compared using a Wilcoxon rank sum test and this data used to create a volcano plot (Fig 1). Of the 618 spots, 93 had a *p*-value < 0.05, and 62 had a greater than 2-fold change. Receiver operator characteristic curves were generated and area under the curve (AuROC) was also calculated for each spot (S1 Fig). The average AuROC was 0.606 with a max AuROC of 0.9167 (spot 2159). Of the 618 spots, 55 had an AuROC ≥0.75 and all of these 55 had *p*-values < 0.05 and were selected to be of interest. The two groups were also compared within Progenesis SameSpots using a t-test, and four more spots with an AuROC < 0.75 but *p*-value < 0.05 were selected to be of interest. Of these combined 59 spots of interest, 49 were chosen for identification by MS/MS based on spot intensity and likelihood of obtaining an identification (Fig 2). Based on these protein spots, sea lions separated into two groups after hierarchical clustering (S2 Fig). Of the protein spots, 19 were elevated in DAT and 30 were lower in DAT. Using values from these 49 spots of interest, principle components analysis was also used to evaluate whether these spots were able to separate DAT and non-DAT in the training set in multidimensional space. The two groups seem to separate, mostly along the first principle component (S3 Fig). Interestingly the highest positive loading contributor for the first principle component was spot 2159 (0.21).

### Spot identification

Gel plugs were digested and of the 49 spots chosen for identification, 34 could be identified with confidence. Some spots contained more than one protein and these data are presented in S1 Table, though the most abundant protein based on protein coverage and number of spectra within each gel plug is presented for simplicity (Table 3). Furthermore, since the data were searched against a multispecies database, some protein IDs were shared across species, and are shown in S1 Table. Protein sequences from positive identifications to entries listed as uncharacterized (from less well characterized species, *e*.*g*., panda, dog, sea lion) were used for Blastp searches and homologous proteins identified if possible, otherwise these were designated ‘unknown’. Eleven spots were associated with ApoE (spot 3535 contained ApoE but this was not the most abundant protein; S1 Table). Spot 3486 had 50 significant peptide matches of nine unique significant peptides, with 28% coverage, whereas the other 10 ApoE spots contained less than five significantly identified tryptic peptides. Furthermore, all 11 spots associated with ApoE were significantly lower in abundance in the DAT group.

**Table 3:** Gel spot protein identifications and statistics.

Spot #	fold Δ	*p*-value	AuROC	UniProt ID	Protein Name
3533	-3.36	0.045	0.717	Q7M2U7	ApoE
3486	-3.19	0.002	0.842	Q7M2U7	ApoE
3442	-3.18	0.003	0.817	Q7M2U7	ApoE
2134	-3.15	0.001	0.863	Q7M2U7	ApoE
3436	-2.76	0.003	0.821	Q7M2U7	ApoE
1409	-2.69	0.002	0.829	F1PDJ9	Unknown
2159	-2.66	0.0001	0.917	Q7M2U7	ApoE
3453	-2.64	0.010	0.779	Q7M2U7	ApoE
3454	-2.63	0.005	0.804	Q7M2U7	ApoE
3452	-2.55	0.008	0.788	Q7M2U7	ApoE
2451	-2.42	0.005	0.800	E2QYU2	Clusterin (ApoJ)
3446	-2.20	0.006	0.796	Q7M2U7	ApoE
2721	-2.04	0.001	0.846	G1LGY8	Immunoglobulin J chain
2583	-2.00	0.015	0.763	Q7M2U7	ApoE
3465	-1.96	0.009	0.783	D2HC79	ApoA-IV
3536	-1.96	0.013	0.767	G1LGY8	Unknown
3501	-1.89	0.004	0.813	E2QYB2	Unknown
3535	-1.80	0.028	0.738	D2HC79	ApoA-IV
3252	-1.38	0.017	0.758	D2HC77	ApoA-1
2452	-1.36	0.015	0.763	G1KZV4	Clusterin (ApoJ)
3396	2.69	0.013	0.767	F1MKC4	Actin
1044	1.93	0.017	0.758	G1MKE1	Carboxypeptidase N subunit 2
3395	1.93	0.017	0.758	F1PQL8	Actin, cytoplasmic 1
1464	1.86	0.017	0.758	A2VE41	EGF-containing fibulin-like extracellular matrix protein 1
3534	1.77	0.003	0.821	G1MAY6	Antithrombin-III
1515	1.71	0.005	0.800	Q2Y099	Vitronectin
3019	1.62	0.019	0.754	G1L6A5	Complement C4-A
1686	1.60	0.004	0.808	G1MJI1	Fibrinogen gamma chain
1651	1.60	0.013	0.767	G1LHM6	Vitamin D-binding protein
3150	1.58	0.021	0.750	F1PH71	Hemoglobin subunit gamma
1628	1.55	0.013	0.767	F1P8G0	Fibrinogen gamma chain
2789	1.50	0.006	0.796	G3X8D7	Glutathione peroxidase
1528	1.41	0.017	0.758	G1LEJ5	Albumin
3173	1.36	0.041	0.721	D2HC77	ApoA-1

Of the 49 spots of interest, 34 could be identified. Fold change (**Δ**) is DAT/non-DAT, with *p*-values calculated using a Wilcoxon rank sum test. The area under the ROC curve (AuROC) is also given. The protein ID (UniProt ID) is of the most abundant protein within each spot. If an uncharacterized protein had no apparent homology to a known protein using Blastp, it is listed as ‘unknown’. Overall, 20 different proteins and their charge forms were identified. A complete version of this table exists in S1 Table.

### Supervised machine learning and model generation

Using the 49 spots of interest, 101 artificial neural networks (ANN) were trained on the training set data. Typically, to avoid overtraining, the ANN with the median AuROC is chosen for validation. Since the median AuROC of these 101 ANNs was 1, an alternative approach was used a termed here as CANN_101_. A Combined ANN (CANN) uses the average output of the ensemble of 101 ANN to generate an ROC curve, which is used to determine a threshold for use on the independent test set. In addition to generating models using protein spot intensity, clinical parameters were also included in model generation. Since data from previous studies indicated that eosinophil counts may be a classifier of DAT [5, 26, 27, 32] and because spot 2159 was the strongest single predictor and was identified as ApoE, we generated a CANN_101_ model using spot 2159 data and eosinophil counts. Likewise, we chose the spot with the highest confidence identification associated with it (spot 3486, ApoE) and combined it with spot 2159, and eosinophil counts in different combinations to generate additional CANN_101_ ensemble models. Next we used these three variables to train a categorical classification tree, a random forest, *k*-nearest neighbor model, and a support vector machine.

### Model qualification with investigator-blinded test set

The performance of selected individual classifiers and models trained using the training set was qualified using 2D-GE analysis of the blinded independent test set and the hematological variable eosinophil count (Table 4). The 20 individuals in the independent test set were selected from plasma taken at admission from seven DAT and 13 non-DAT that were frequency matched by gender (*p* = 0.5 using a one-sided Fisher’s exact test; Table 5). The non-DAT test set had diverse etiology similar to the training set. Both non-DAT and DAT groups had similar hematology except that white blood cell count and segmented neutrophil counts were lower in the DAT group (Table 6). In both the test set and training set eosinophil counts were lower in the non-DAT group.

**Table 4:** Statistical performance of models to discriminate DAT cases in an independent test set.

Input Data	Model Type	Threshold	Sensitivity Test Set	Specificity Test Set	trAuROC	trSens	trSpec
Spot 2159	ROC curve	minMC	100%	38.5%	0.917	91.7%	85.0%
Spot 3486	ROC curve	minMC	85.7%	69.2%	0.842	75.0%	90.0%
Spot 3486	ROC curve	opT	100%	61.5%	0.842	83.3%	80.0%
Eosinophil Count	ROC curve	minMC	57.1%	92.3%	0.759	72.7%	76.5%
49 spots	CANN_101_	minMC	100%	23.1%	1.000	100%	100%
2159 + 3486	CANN_101_	minMC	100%	61.5%	1.000	100%	100%
2159 + Eos	CANN_101_	minMC	85.7%	84.6%	1.000	100%	100%
3486 + Eos	CANN_101_	minMC	85.7%	84.6%	1.000	100%	100%
2159 + 3486 + Eos	CANN_101_	minMC	85.7%	76.9%	1.000	100%	100%
2159 + 3486 + Eos	CT	NA	85.7%	84.6%	NA	90.9%	100%
2159 + 3486 + Eos	*k*-NN	NA	85.7%	69.2%	NA	72.7%	89.5%
2159 + 3486 + Eos	RF	NA	85.7%	69.2%	NA	100%	100%
**2159 + 3486 + Eos**	**SVM**	**NA**	**85.7%**	**92.3%**	**NA**	**81.8%**	**94.7%**

An AuROC for the test set was not determined since this set was only used to qualify models derived from the training set. The top performing model, SVM using spots 2159, 3486 and eosinophil counts, is bolded. NA, Not Applicable. *Abbreviations: ROC curve, receiver operator characteristic curve; CANN, combined artificial neural networks; CT, classification tree; k-NN, k-nearest neighbor; RF, random forest; SVM, support vector machine; minMC, minimum mis-classified; opT, optimum threshold; AuROC, area under the ROC curve; tr prefix, values are from training set.*

**Table 5:** Test set characteristics table.

	Total	DAT	Non-DAT
**Total**	20	7	13
male	10	3 (43%)	7 (54%)
female	10	4 (57%)	6 (46%)
**Age**			
*pup*	0	0 (0%)	0 (0%)
*yearling*	2	0 (0%)	2 (15%)
*juvenile*	4	1 (14%)	3 (23%)
*subadult*	6	2 (29%)	4 (31%)
*adult*	8	4 (57%)	4 (31%)
**Euthanasia/Death**	9	7 (100%)	2 (15%)
**Primary Etiology (non-DAT)**			
*leptospirosis*		-	3
*trauma*		-	3
*carcinoma*		-	0
*malnutrition*		-	4
*pneumonia*		-	3

Descriptive data of sea lions in the test set.

**Table 6:** Hematological parameters of the test set.

	DAT	non-DAT	
Parameter	Avg ± S.D. (n)	Avg ± S.D. (n)	*p*-value
WBC (10^3^/mm^3^)	13.0 ± 4.5 (n = 7)	21.0 ± 8.9 (n = 13)	0.014*
RBC (10^6^/mm^3^)	4.3 ± 0.5 (n = 7)	4.2 ± 0.8 (n = 11)	1.000
HGB (g/dL)	15.0 ± 2.1 (n = 7)	14.3 ± 3.2 (n = 11)	0.412
HCT (%)	44.0 ± 4.7 (n = 7)	41.6 ± 9.3 (n = 13)	0.428
PLT (fL)	475.3 ± 182.8 (n = 7)	408.0 ± 164.7 (n = 11)	0.860
MCV (pg)	103.3 ± 2.0 (n = 7)	99.9 ± 5.0 (n = 11)	0.079
MCH (g/dL)	35.0 ± 2.1 (n = 7)	33.7 ± 1.5 (n = 11)	0.099
MCHC (10^3^/mm^3^)	33.8 ± 2.0 (n = 7)	33.7 ± 0.8 (n = 11)	0.218
RDW (%)	15.7 ± 1.1 (n = 7)	15.8 ± 0.6 (n = 11)	0.740
MPV (fL)	7.7 ± 0.7 (n = 7)	8.0 ± 0.7 (n = 11)	0.441
Seg (#/mm^3^)	9564 ± 4165 (n = 7)	16769 ± 8229 (n = 13)	0.032*
Band (#/mm^3^)	192 ± 353 (n = 7)	1118 ± 1637 (n = 13)	0.089
Lymph (#/mm^3^)	2027 ± 1118 (n = 7)	2381 ± 1075 (n = 13)	0.219
Mono (#/mm^3^)	176 ± 205 (n = 7)	548 ± 559 (n = 13)	0.077
Eos (#/mm^3^)	996 ± 637 (n = 7)	153 ± 260 (n = 13)	0.005*

Blood data is based on analysis of blood drawn the same day as samples used for analysis. Complete blood count analyses of the 20 samples used in the test set are listed. Some parameters were not measured for all individuals and are indicated by ‘n’. Hematological parameters were compared between chronic domoic acid toxicosis (DAT) and non-DAT groups using a Wilcoxon rank-sum test with normal approximation, and significance (*p*<0.05) is highlighted by an ‘*’. *Abbreviations: WBC, white blood cell count; RBC, red blood cell count; HGB, hemoglobin; HCT, hematocrit; PLT, platelet; MCV, mean corpuscular volume; MCH, mean corpuscular hemoglobin; MCHC, mean corpuscular hemoglobin concentration; RDW, red cell distribution width; MPV, mean platelet volume; Seg, segmented neutrophils; Band, band cells; Lymph, lymphocytes; Mono, monocytes; Eos, eosinophils.*

Of the single classifiers evaluated, spot 3486 and eosinophil counts were the best discriminators of chronic DAT, achieving the best balance of sensitivity and specificity. Ensembles of artificial neural networks trained using all 49 protein spots consistently over predicted DAT (23.1% specificity), while using just two ApoE spots had better specificity (61.5%). The classification tree using both ApoE spots and eosinophil counts performed well with 85.7% sensitivity and 84.6% specificity (an eosinophil count of greater than or equal to 365 was used as a second step node to discriminate DAT), while a random forest using the same variables achieved lower specificity than the classification tree (69.2% versus 84.6%). The *k*-nearest neighbor model performed the same as the random forest, though the incorrectly classified individuals were different. Two combinations of variables with CANN_101_ had the same performance, ApoE spot 2159 plus eosinophil counts and ApoE spot 3486 plus eosinophil counts, though these models only performed as well as the classification tree. The best overall performer was the support vector machine using both ApoE spots and eosinophil counts, which only mis-predicted one non-DAT and one DAT individual in the independent test set.

## Discussion

We present here an unbiased proteomic analysis of California sea lion plasma in an effort to identify markers of chronic DAT. Using 2D-GE, 49 significantly different spots comprising 20 proteins and their isoforms were identified by tandem mass spectrometry. The majority of the protein spots with negative fold-change values (11 of 20) were identified as ApoE; whereas the proteins listed in the positive fold-change group tended to be more evenly distributed amongst common plasma proteins. Perhaps confusing is the fact that so many of the spots were identified as ApoE. When utilizing 2D-GE, any modification of the protein can affect the distribution of the modified protein in the gel because proteins are separated in two dimensions and the migration of the protein is a function of charge and molecular weight. In the case of modification, proteins migrate to multiple spots in the gel and for statistical comparisons, each spot is treated as an independent observation. In the case of ApoE, this protein is o-glycosylated in sea lions and migrates as two distinct bands when analyzed by one dimension SDS-PAGE [33]. In humans the complexity of ApoE glycoforms has been recently assessed utilizing mass spectrometry. Evidence for multiple glycans at threonine 194 and serine 209 correlates with a diverse distribution of ApoE analyzed by 2D-GE suggesting that modification of ApoE is far more complex than previously described [34]. Based on our findings of multiple charge forms of ApoE in California sea lion serum, it is likely that a similar level of ApoE glycan complexity exists in marine mammals. Although the significance of reduced abundance of ApoE charge forms in the California sea lion is unknown, a reduction in glycoforms of ApoE has been correlated with increased amyloid beta peptide 42 accumulation in a mouse model of Niemann-Pick Disease where a “simplification” of ApoE glycosylation appears to correspond with neurodegeneration via impaired cholesterol trafficking [35]. Given the over-representation of ApoE in our differentially abundant results and that chronic DAT is neurodegenerative, it is probable that changes in ApoE charge form distribution are important in the progression to chronic DAT.

There are numerous reports surrounding ApoE in the context of neurological disorders. A study in transgenic mice demonstrated that human ApoE2/3 forms help to maintain the blood brain barrier when murine ApoE was deleted [36]. Early reports of ApoE deficient mice showed increased vascular permeability in the brain lending credence to the importance of ApoE in neurological disorders. ApoE also plays an important role in transporting cholesterol to cells for cell repair and steroid synthesis [37]. Mice deficient for ApoE exhibit higher plasma cholesterol and lower esterified cholesterol stores in adrenocortical cells compared to controls demonstrating that ApoE is important for the maintenance of esterified cholesterol stores in the adrenal glands and suppression of corticosterone [38, 39]. Sea lions with DAT have been reported to have depressed cortisol levels despite ACTH levels that are no different than controls [27], suggesting that adrenal insufficiency is likely a valid clinical sign of DAT. Because sea lion samples included in the adrenal insufficiency study were also included in this study, it is possible, but speculative, that DAT through the axis of ApoE could lead to metabolic disturbances in cholesterol metabolism in the adrenal gland. Further studies on the effects of specific ApoE glycoforms are necessary to determine whether these proteins can modify cholesterol storage in adrenal glands. These hypotheses are interesting, but the role of ApoE in the context of DAT remains a worthwhile question if the protein results are validated in a larger study.

In addition to providing insight into the mechanism of chronic DAT, these results also identify a candidate biomarker panel of chronic DAT. Of the 11 protein spots associated with ApoE, two ApoE spots were chosen for further investigation: the spot with the highest confidence ApoE identification (spot 3486) and the strongest single predictor (spot 2159). Using an independent test set to qualify these protein spots as diagnostic of chronic DAT, it was demonstrated that excellent sensitivity could be achieved using charge forms of ApoE, but these markers over-classified DAT (*i*.*e*., low specificity). To improve this performance, eosinophil counts were included in the biomarker panel since eosinophil counts have been shown to be increased in DAT [5, 26, 27, 32], and in our study were actually found to perform reasonably well to discriminate DAT from non-DAT. In order to develop a classifier comprised of a combination of these three markers, supervised machine learning approaches were employed to identify predictive patterns in multidimensional space. The best performing classifier was a support vector machine trained on two ApoE charge forms and eosinophil counts. This model achieved high sensitivity (85.7% with 92.3% NPV) and good specificity (92.3% with 85.7% PPV), only mis-predicting one ‘healthy’ and one DAT individual. The inclusion of eosinophil counts mitigated the over-classification error of ApoE while maintaining high sensitivity. Although a larger data set is required to set an accurate eosinophil threshold for DAT, the classification tree model used eosinophil counts greater than or equal to 365 to differentiate individuals with low ApoE. A similar threshold cannot be determined from the support vector machine since the hyperplane is nonlinear. Typically blood counts are used for narrowing the diagnosis differential, but are not included in biomarker panels because the data is thought to be too generic or not mechanistically related. In this data and other published studies of blood count data in DAT it is evident that high eosinophil counts is a risk marker of DAT [5, 26, 27, 32]. Likewise, our results demonstrate the utility of eosinophil counts when combined with a protein biomarker, and highlights the potential use of blood variables in discriminating DAT in sea lions.

Despite recent advances in developing minimally invasive test for DAT, there is still a clear need for a rapid, robust and accurate diagnostic assay. Currently biomarkers of DAT have not been translated into the clinical setting though both a behavioral test [8] and a serum peptide MALDI profiling assay [26] have been described. The simplicity of the two component candidate biomarker panel described herein lends itself to deployment as a point of care assay for validation and optimization in large populations with defined endpoints once the target ApoE forms are identified and a standardized test is created. Moreover, the results demonstrate promise for future studies combining blood biomarkers, clinical measures and other disparate patient variables in multidimensional space to create more robust classifiers. Lastly, even though this study focused on chronic DAT, we postulate that these markers may be useful for diagnosing sea lions exhibiting clinical symptoms of toxicosis on the continuum of acute to chronic DAT.

## Limitations

There are several limitations to this study. The test set upon which biomarkers were validated is very small and may not accurately reflect the total sea lion population. The small training and test sets emphasize the need for larger training and validation. Regardless, the performance of our rationally chosen variables to construct non-linear models to classify an independent test set highlights the potential use of these variables in discriminating DAT in sea lions. Additionally, pregnant females were specifically excluded from this study to limit plasma protein variability within groups and therefore our results are not applicable to that portion of the sea lion population. Also, the non-DAT group included animals housed at The Marine Mammal Center for differing periods of time. Captivity has been shown to elevate levels of haptoglobin in sea lions [40], exemplifying the dynamic nature of serum proteins in high stress environments. Whether ApoE levels change with captivity or treatment is unknown, but should be considered in ensuing studies. Furthermore, specific ApoE variant levels were measured by spot intensity after 2D gel electrophoresis. The levels of ApoE variant are not standardized measurements reported in standard units, therefore, clinically meaningful thresholds could not be determined. Future large-scale studies will require both assay and marker validation against traceable standards for implementation in the clinic. Lastly, the proteomic data was analyzed using a database comprised of phylogenetically similar species to, and including, the California sea lion since there are currently less than 90 documented California sea lion proteins in the UniProt Protein Knowledge Base. In the proteomic results only one protein, ApoE, was identified based off a California sea lion specific sequence, whereas 12 proteins were identified using the recently completed *Ailuropoda melanoleuca* database, and only six from the *Canis familiaris* database. Since protein identification scoring from tandem mass spectrometry data is intolerant of single amino acid changes, it is likely either that at the molecular level California sea lions are more similar to pandas than dogs, the current dog database is not as well developed as the panda, or that this is too small of a query size to make meaningful phylogenetic conclusions. Regardless, these results highlight an acceptable current approach to analyzing California sea lion proteomic data while a genome is being completed. Ongoing proteomic analysis of fluids (plasma, serum, and cerebrospinal fluid) from California sea lions will utilize standard shotgun proteomic approaches. These studies along with the continued development of a genome will improve our ability to mine the molecular milieu for markers of disease in the California sea lion.

## Supporting Information

S1 Fig AuROC Distribution of 618 spots in the training set. Bins are greater than value immediately lower on the axis. Ex. 1 AuROC>0.9.
(TIF)

S2 Fig Hierarchal clustering of the training set using data from 49 selected spots. Data were first standardized along spot data by transforming data such that the mean for each spot equals zero, and one standard deviation unit is 1, either positive or negative. The scale given reflects this transformation. Data were clustered along individuals, producing spot-clustered data.
(TIF)

S3 Fig Principle components analysis enhanced with centroids using data from 49 spots. Average PC1 and PC2 scores are solid points, with hollow points being individuals.
(TIF)

S1 Table Full Table of Protein Identification of 34 gel plugs. Of the 49 spots of interest, 34 could be identified. Fold change (Δ) is DAT/non-DAT, with *p*-values calculated using a Wilcoxon rank sum test. The area under the ROC curve (AuROC) is also given. The protein ID (UniProt ID) is of the most abundant protein within each spot. If an uncharacterized protein had no apparent homology to a known protein using Blastp, it is listed as ‘unknown’. The score is the Mascot protein score. The ‘Matches’ column shows the number of spectra that were matched to a peptide (and the number of significant matches). The ‘Sequences’ column is the number of spectra matched to a distinct peptide sequence (and the number of significant matches). emPAI is exponentially Modified Protein Abundance Index, which is a relative quantification related to protein coverage. Lastly, the ‘Description’ column is the protein description from UniProt which includes the protein name, species (OS), gene symbol, if applicable (GN).
(XLS)
