# Supplementary figures and images for: Proteomic Analysis of Plasma from California Sea Lions (Zalophus californianus) Reveals Apolipoprotein E as a Candidate Biomarker of Chronic Domoic Acid Toxicosis
Source: PLoS One. 2015 Apr 28;10(4):e0123295. doi: 10.1371/journal.pone.0123295 (PMC4412824; doi:10.1371/journal.pone.0123295)

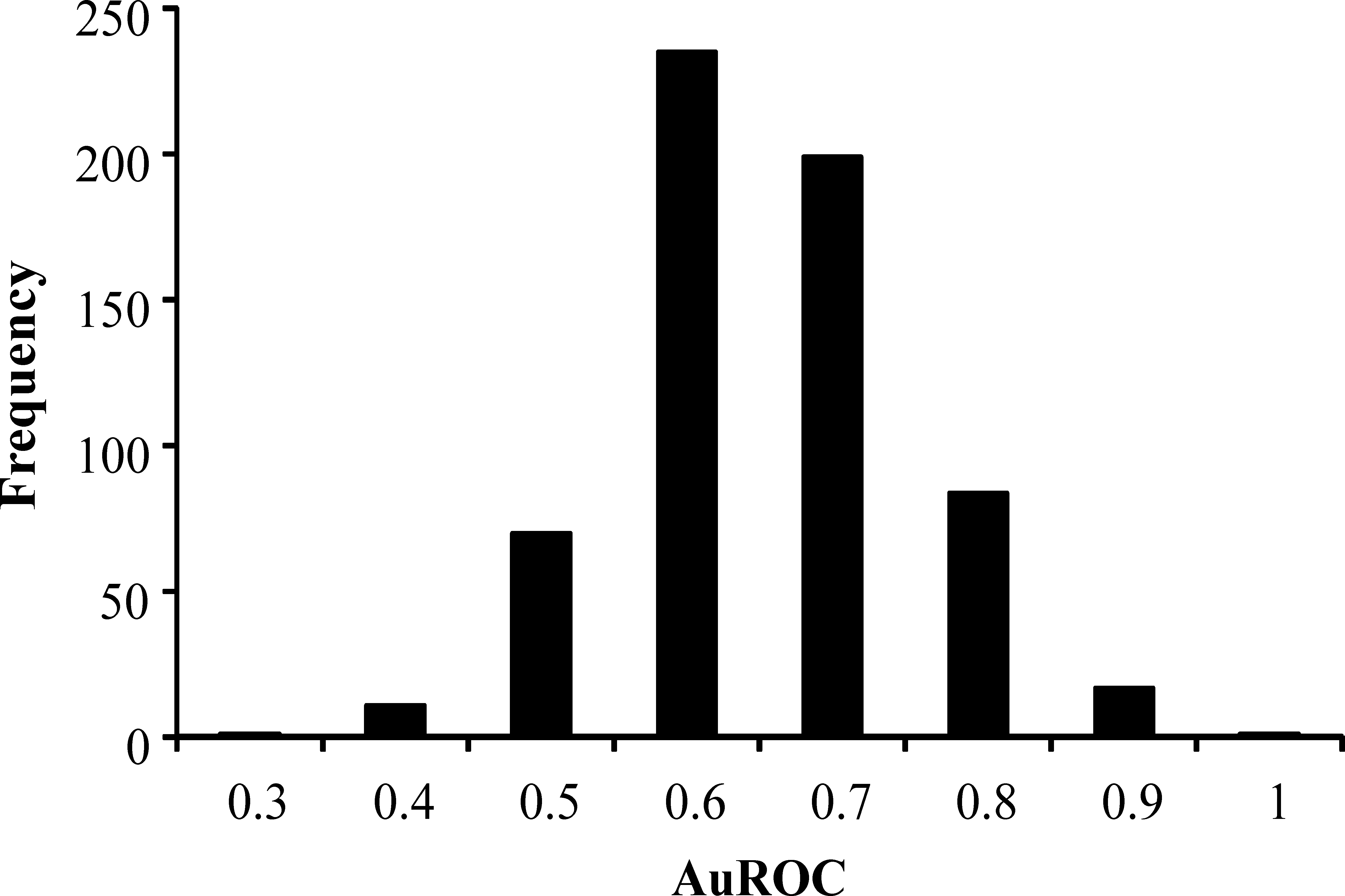

Supplement: S1 Fig — Bins are greater than value immediately lower on the axis. Ex. 1 AuROC>0.9. (TIF) [file pone.0123295.s001.tif]

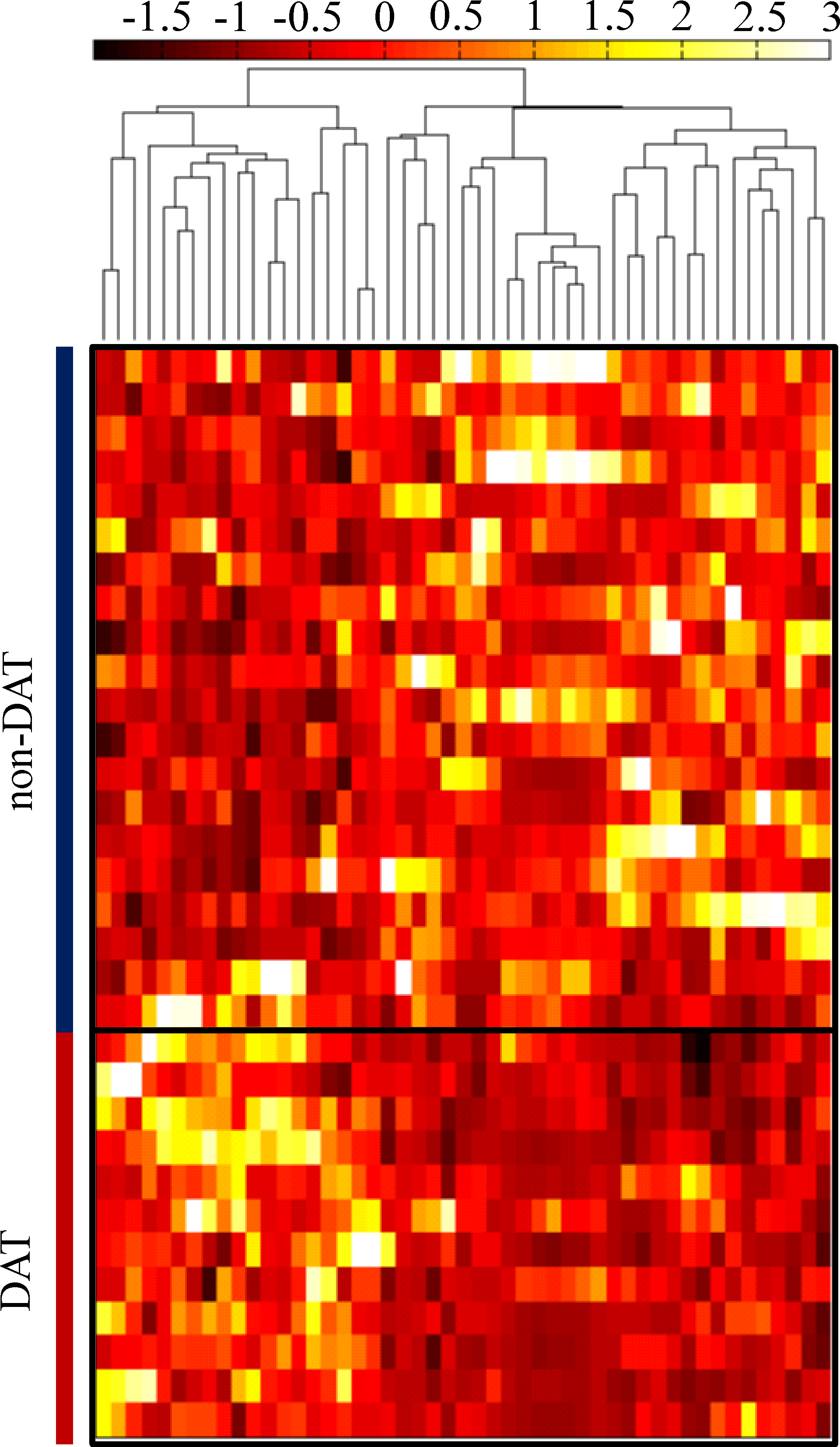

Supplement: S2 Fig — Data were first standardized along spot data by transforming data such that the mean for each spot equals zero, and one standard deviation unit is 1, either positive or negative. The scale given reflects this transformation. Data were clustered along individuals, producing spot-clustered data. (TIF) [file pone.0123295.s002.tif]

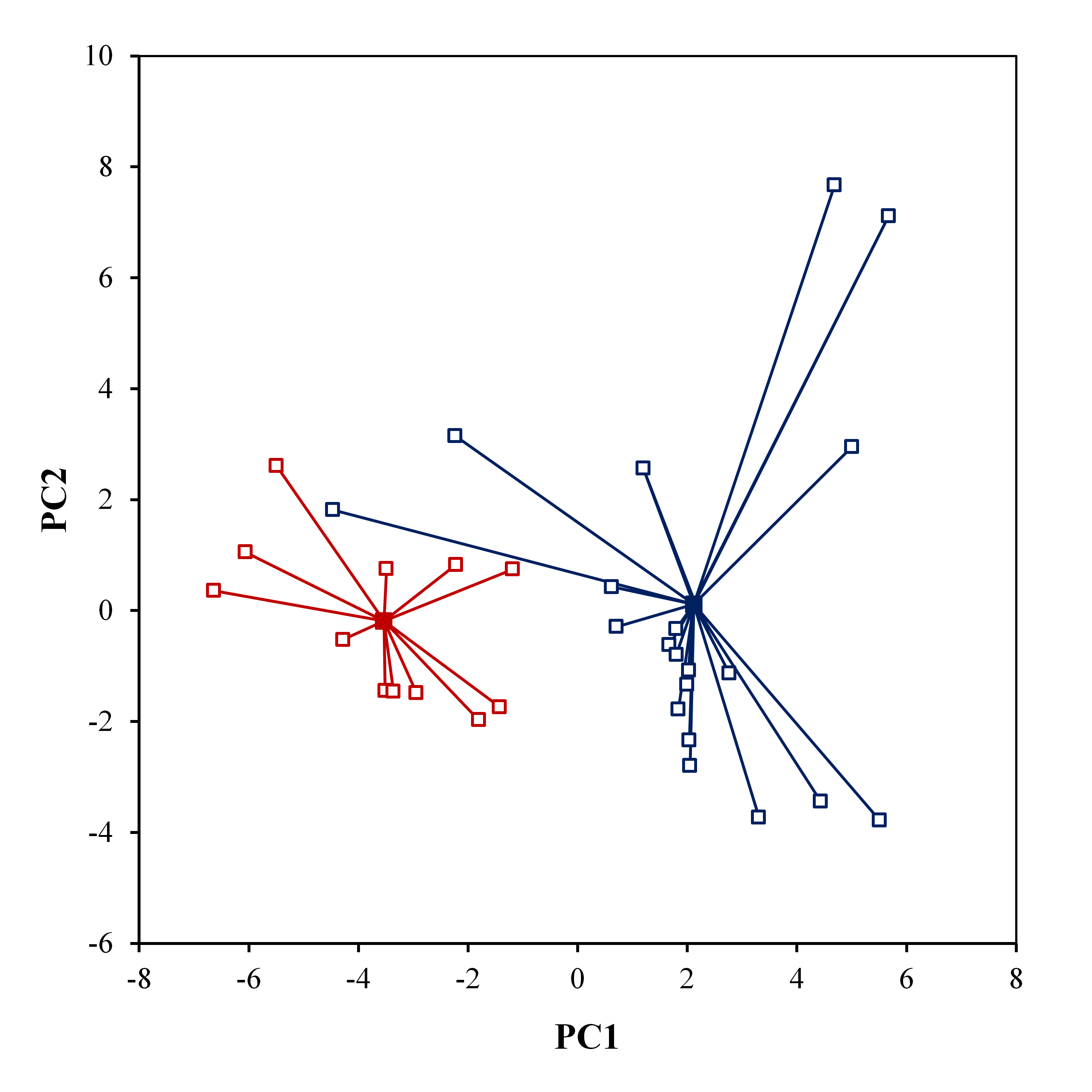

Supplement: S3 Fig — Average PC1 and PC2 scores are solid points, with hollow points being individuals. (TIF) [file pone.0123295.s003.tif]
